# Effect of Porcine Colostral Exosomes on T Cells in the Peripheral Blood of Suckling Piglets

**DOI:** 10.3390/ani12172172

**Published:** 2022-08-24

**Authors:** Hiroto Miura, Itsuki Jimbo, Machi Oda, Michiko Noguchi, Kiyonori Kawasaki, Mayuko Osada-Oka, Takamitsu Tsukahara, Ryo Inoue

**Affiliations:** 1 Laboratory of Animal Science, Setsunan University, Hirakata, Osaka 573-0101, Japan; 2 Laboratory of Animal Science, Kyoto Prefectural University, Kyoto, Kyoto 606-8522, Japan; 3 Laboratory of Theriogenology, Azabu University, Sagamihara, Kanagawa 252-5201, Japan; 4 Graduate School of Agriculture, Kagawa University, Kita, Kagawa 761-0795, Japan; 5 Food Hygiene and Environmental Health, Faculty of Life and Environmental Sciences, Kyoto Prefectural University, Kyoto, Kyoto 606-8522, Japan; 6 Kyoto Institute of Nutrition & Pathology, Tsuzuki, Kyoto 610-0231, Japan

**Keywords:** piglet, colostrum, exosome, T cells, early post-natal development

## Abstract

**Simple Summary:**

Exosomes in porcine colostrum have gained attention as the possible key compounds involved in the growth and/or development of suckling piglets. In this study, peripheral blood mononuclear cells (PBMCs) from suckling piglets were cultured with or without milk-derived exosomes (control) in vitro. Porcine colostral exosomes increased the proportion of cytotoxic T (Tc) cells, while this phenomenon was not observed in PBMC whose endocytosis was inhibited. Moreover, exosome-treated PBMCs had a higher cytokine IL-2 concentration in the culture supernatant than the control. The present study demonstrated that porcine colostral exosomes could increase the Tc cell proportion in the peripheral blood of a suckling piglet, with the underlying mechanism believed to be the stimulation of IL-2 production in PBMCs via endocytosis.

**Abstract:**

Growing evidence indicates that porcine colostral exosomes may contribute to the healthy development of piglets. Here, we evaluated in vitro the effect of porcine milk-derived exosomes, in particular colostral exosomes, on T cells in the peripheral blood of suckling piglets. A total of seven sows and thirteen suckling piglets were used. Peripheral blood mononuclear cells (PBMCs) from suckling piglets were cultured with or without milk-derived exosomes (control). Using flow cytometry, the proportion of each T cell subset in cultured PBMCs was analyzed three days post-incubation. PBMCs cultured with porcine colostral exosomes had a higher proportion of CD3^+^CD4^−^CD8^+^ T cells (cytotoxic T cells; Tc) than the control. However, exosomes induced no increase in the Tc cell population in PBMC whose endocytosis was inhibited. We further measured the concentrations of cytokines in the culture supernatant. Exosome-treated PBMCs had a higher cytokine IL-2 concentration than the control. The present study demonstrated that porcine colostral exosomes could increase the Tc cell proportion in the peripheral blood of suckling piglets, with the underlying mechanism believed to be the stimulation of IL-2 production in PBMCs via endocytosis. Moreover, our results suggested that porcine colostral exosomes were involved in the development of cellular immunity in suckling piglets.

## 1. Introduction

To grow healthy, ingesting porcine colostrum is crucial for suckling piglets [1]. The porcine colostrum plays an essential role in providing piglets with nutrients and growth factors that promote organ development [2]. In addition, for newborn piglets, porcine colostrum is a vital source of passive immunity that would not otherwise be available due to the unique porcine epitheliochorial placenta. The porcine placenta prevents the provision of maternal immunological components to the fetus [2]. The immunological components in porcine colostrum, such as immunoglobulin, are transferred to the bloodstream of piglets via absorption in their digestive tract and act against bacterial and viral infection [3]. Moreover, the porcine colostrum has also been reported to stimulate the development of the piglet’s mucosal and systemic immune system [4].

The porcine colostrum is mainly composed of protein, carbohydrates, lipids, lesser minerals, vitamins, leukocytes, somatic cells, bacteria, and exosomes [1]. In the past decade, exosomes in porcine colostrum have gained attention as the possible key compounds involved in the growth and/or development of suckling piglets [5,6]. Exosomes are nanosized, endosomal-derived membrane micro-vesicles, containing functional molecules such as mRNA, microRNA, proteins, and lipids [7]. Exosomes can mediate cell–cell communication and are involved in various physiological cellular processes including the immune response, signal transduction, and antigen presentation [7,8]. Previous studies showed that exosomes in porcine colostrum were transferred to piglets in the same manner as other immune-related components and growth factors [5,6,9]. For instance, Gu et al. [6] reported that porcine colostral exosomes are enriched with immune-related microRNA, whose concentration in serum was higher in newborn piglets fed colostrum than in those fed mature milk. Chen et al. [5] suggested that exosomes in the porcine milk stimulated the proliferation of small intestinal epithelial cells in vitro. In addition, Zeng et al. [10] demonstrated that exosomes in porcine milk (collected 3–5 days post-parturition) promoted intestinal immunoglobulin production by stimulating the expression of polymeric immunoglobulin receptors both in vitro and in vivo. These reports seemed to imply that porcine colostral exosomes contributed to the growth and/or development of suckling piglets. Nonetheless, available data on the matter are unsatisfactory.

To expand our understanding of the functions of porcine milk-derived exosomes, especially colostral exosomes, in the present study, our aim was to investigate their involvement in the development of the immune system of piglets. To evaluate the effects of porcine milk-derived exosomes on blood T cells, we conducted an in vitro co-culture of milk-derived exosomes and mononuclear cells from the peripheral blood (PBMCs) of suckling piglets. In the present study, T cells were singled out because they can orchestrate both humoral and cellular immune responses, which are crucial in immunity.

## 2. Materials and Methods

### 2.1. Sample Collection

The animal experiments were conducted in accordance with the guidelines of the Kyoto Prefectural University under the approved protocol KPU240206R-C. To employ a sufficient number of animals (Table 1), the present study was carried in three independent experiments (identified as Experiments 1, 2 and 3). A total of 7 sows (Large White × Landrace, *n* = 3; Landrace, *n* = 4; parity 5.1 ± 2.3; 11.0 ± 3.5 piglets/litter) and 13 piglets (Large White × Landrace × Duroc, *n* = 9; Large White × Landrace, *n* = 4; 25.5 ± 5.0 days of age) were used. There were no dam–litter relationships between sows and piglets. Each experiment was carried out using 2–3 sows and 4–5 piglets (Table 1). Animals were reared at animal facilities in Azabu University (Kanagawa, Japan), Kagawa University (Kagawa, Japan) and Kyoto Prefectural University (Kyoto, Japan). Sows used were not fed immune-stimulant/modulation additives during lactation. Colostral samples were collected from individual sows during parturition and stored at −80 °C until further use. In Experiment 2, mature-milk samples were also collected from the same sows whose colostrum were taken (Sow 3 and 4), three weeks post-parturition. Peripheral blood was drawn from the abdominal or jugular veins of piglets and collected into Venoject II heparinized, vacuum blood collection tubes (Terumo Medical Corp., Tokyo, Japan). The blood samples were kept at 4 °C and transported to the designated laboratory, where they were processed within 24 h post-collection.

### 2.2. Isolation of Exosomes from Porcine Colostrum and Mature Milk

**Figure 1:**
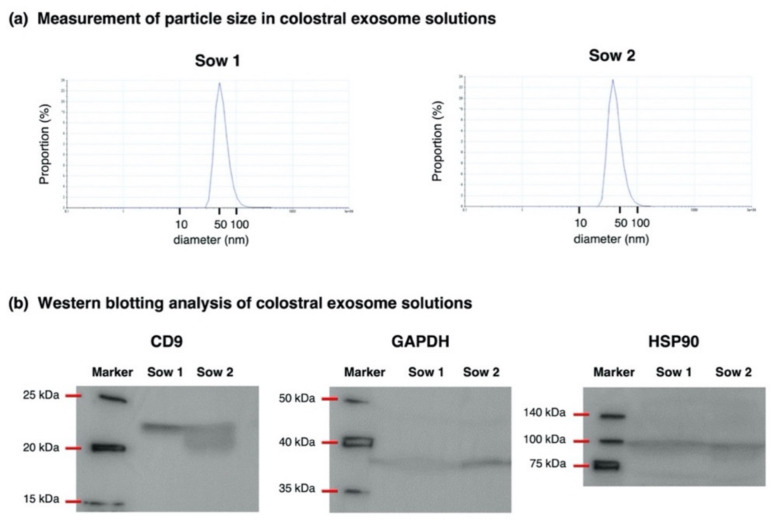
Measurement of the particle size (**a**) and Western blotting analysis (**b**) for the validation of the isolation of exosomes from porcine colostrum. (**a**) The particle size of exosomes was measured in two solutions using a Zetasizer Nano apparatus (Malvern Panalytical Ltd., Malvern, UK). (**b**) Using Western blotting, the exosome solutions were screened for exosome marker protein CD9, glyceraldehyde-3-phosphate dehydrogenase (GAPDH) and heat shock protein 90 (HSP90).

Colostral and mature-milk samples were diluted with phosphate buffered saline (PBS) (1:1) and the solutions centrifuged at 1500× *g* at room temperature for 20 min. The resulting supernatants were serially filtered using 0.8 µm-, 0.45 µm- and 0.2 µm-mesh syringe filters (PALL, Port Washington, NY, USA). The filtered solutions were then centrifuged at 100,000× *g* at 4 °C for 3 h, and afterwards, the supernatants were discarded. The retrieved pellets were suspended in PBS and centrifuged again at 100,000× *g* at 4 °C for 1.5 h. Again, supernatants were discarded and the retrieved pellets, likely containing exosomes, were suspended again in PBS. In Experiment 1, based on the particle size and expression of exosome marker proteins [CD9, glyceraldehyde-3-phosphate dehydrogenase (GAPDH) and heat-shock protein 90 (HSP90)], we confirmed the isolation of exosomes (Figure 1). To determine particle size and intensity, the exosomes retrieved by ultracentrifugation were analyzed using a Zetasizer Nano apparatus (Malvern Panalytical Ltd., Malvern, UK). For the Western blotting analysis, exosomes were lysed with RIPA buffer [10 mM Tris-HCl, pH 7.4; 5 mM EDTA pH 8.0; 3.5 mM SDS; 1% TritonX-100; 24 mM sodium deoxycholate; 199 nM Nα-tosyl-L-phenylalanine chloromethyl ketone; 100 nM tsyl-L-lysyl-chloromethane hydrochloride; 150 mM NaCl; 0.05 M NaF; 25 mM β-glycerophosphate pentahydrate; 1 mM Na_3_VO_4_; and 1 cOmplete Mini tablet (Roche, Basel, Switzerland)/10 mL]. Samples were suspended in sodium dodecyl sulphate (SDS) buffer (30 mM Tris-HCl; pH 8.8, 1% SDS; 1% 2-mercaptethanol; 3.67% glycerol; and 0.017% bromophenol blue) and heated at 95 °C for 5 min. Samples were placed onto 12.5% polyacrylamide gels and immunoblotted against HSP90 (1:1000, Becton Dickinson), GAPDH (1:1000, Cell Signaling Technology, Danvers, MA, USA) and CD9 [1:1000, Abcam; diluted with Can Get Signal (Toyobo, Osaka, Japan) or 0.5% skim milk (Morinaga, Tokyo, Japan)]. Blotted proteins were visualized with horseradish peroxidase (HRP)-conjugated goat anti-rabbit IgG (Dako, Santa Clara, CA, USA) or HRP-conjugated goat anti-mouse IgG and Immobilon Western Chemiluminescent HRP substrate (Millipore, Billerica, MA, USA). The amount of protein detected by antibodies was measured using a computerized image analysis system (Lumino Graph 1; Atto, Osaka, Japan).

### 2.3. Isolation of Piglet PBMCs

Blood samples were diluted with PBS (1:2) and Percoll^®^ density-gradient centrifuged (d = 1.077) (GE Healthcare, Tokyo, Japan) at 800× *g* at room temperature for 30 min. Retrieved red blood cells were further lysed with a blood red cell lysing solution (0.15 M NH_4_Cl; 10 mM KHCO_3_; and 0.1 mM Na_2_EDTA, pH 7.4). PBMCs were washed with PBS and suspended in a culture medium (RPMI1640; Nacalai Tesque, Kyoto, Japan) with 10% fetal calf serum (Sigma Aldrich, Dorset, UK), 100 U/mL of penicillin and 100 μg/mL of streptomycin (Nacalai Tesque). Cell counting was performed using a TC20^TM^ automated cell counter (Bio-Rad, Tokyo, Japan).

### 2.4. Dynamin Inhibition in Piglet PBMCs

In Experiment 2, for the inhibition of dynamin, a fraction of the isolated PBMCs (1 × 10^5^ cells) was treated with 80 µM Dynasore hydrate (3-Hydroxy-2-naphthalenecarboxylic Acid; Sigma Aldrich, Dorset, UK) for 1 h prior to the co-culturing with exosomes.

### 2.5. Culture of Piglet PBMCs with Milk-Derived Exosomes

Using the above-mentioned culture medium, the density of piglet PBMCs (including Dynasore-treated PBMCs) was adjusted to 1 × 10^6^ cells/mL. Upon the addition of either 5 µL of PBS (control) or 5 µL of milk-derived exosome solution, 100 µL of PBMCs (1 × 10^5^ cells) was incubated at 37 °C for 3 days, in a 5% CO_2_ condition. As noted in Table 1, we performed co-culturing of piglet PBMCs with porcine colostral exosomes in 28 combinations and with mature-milk exosomes in 6 combinations. All cultures were conducted in quadruplicate.

### 2.6. Analysis of Cell Populations in Piglet PBMCs Using Flow Cytometry

Three days post-incubation, PBMC samples were centrifuged at 800× *g* at room temperature for 3 min, and the supernatants were discarded. Next, PBMC pellets were stained with mouse monoclonal antibodies against porcine CD3 [clone PPT3; R-Phycoerythrin (PE)/Cyanine (Cy)-5-conjugated; Abcam, Tokyo, Japan], CD4a (clone 74-12-4; PE-conjugated; Becton Dickinson, Tokyo, Japan) and CD8a [clone 76-2-11; fluorescein isothiocyanate (FITC)-conjugated; Abcam]. The stained PBMCs were then loaded onto an AccuriTMC6 flow cytometer (Becton Dickinson, Franklin Lakes, NJ, USA) and analyzed using C SamplerTM software (Becton Dickinson). Lymphocytes were identified based on particle size (forward scatter and side scatter). Within the lymphocytes, CD3^+^ cells were identified as T cells and further classified as follows: CD4^+^CD8^−^ T cells, helper T (Th) cells; CD4^−^CD8^+^ T cells, cytotoxic T (Tc) cells; CD4^+^CD8^+^ T cells, double positive T (DP-T) cells; CD4^−^CD8^−^ T cells, double negative T (DN-T) cells. Representative images of the gating strategy are shown in Figure S1. The population of each T cell subset was expressed as a percentage of total T cells (CD3^+^ lymphocytes). Before staining with antibodies, the proportion of live cells was evaluated using trypan blue and cell counter TC20^TM^.

### 2.7. Measurement of IFN-γ, IL-2 and IL-12 in Culture Medium

In Experiment 3, three days post-incubation, three combinations of co-culture showing the most marked increase in the Tc cell population in exosome-treated PBMCs were selected and the concentrations of interferon-gamma (IFN-γ), interleukin-2 (IL-2) and interleukin-12 (IL-12) in culture supernatants were measured. The concentrations of IFN-γ, IL-2 and IL-12 were measured using a Porcine IFN-gamma ELISA kit (Thermo Fisher Scientific, Waltham, MA, USA), a Porcine IL-2 ELISA kit (ABclonal Technology, Woburn, MA, USA), IL-12 (IL-12A) and a Porcine ELISA kit (Thermo Fisher Scientific), as per the manufacturers’ instructions. For all analyses, absorbance at 450 nm was measured using a microplate reader (iMark; Bio-Rad).

### 2.8. Statistical Analysis

A mixed model analysis with four factors, namely, exosome treatment, genetic background, age and rearing environment (farm) was performed in advance by JMP pro v.15.1.0 (SAS Institute Inc., Cary, NC, USA) and found the latter three factors did not affect the results of the exosome treatment on T cell populations. Therefore, to be simple, genetic background, age and rearing environment were not considered as variables for the statistical analysis in this study. First, the normality of the data was assessed using the Shapiro–Wilk test. Each T cell subset proportion was compared in PBMCs cultured with or without milk-derived exosomes, using the Wilcoxon singed-rank test as the data were not normally distributed. The concentrations of IFN-γ, IL-2 and IL-12 in PBMCs cultured with or without colostral exosomes were compared, using the paired *t*-test as the data were normally distributed. Data were considered statistically significant if *p* < 0.05. All statistical comparisons were conducted using R software (v.3.6.2; https://www.r-project.org).

## 3. Results

### 3.1. Validation of Exosome Isolation from Porcine Colostrum

For the exosome solutions used in Experiment 1, the isolation of the exosome from the porcine colostrum was validated by measuring the particle size and confirming the existence of the exosome marker protein by a Western blotting analysis (Figure 1; whole blot images are shown in Figure S2). Particle size in exosome solutions ranged from 10 to 100 nm and showed a peak at approximately 50 nm, which was consistent with the size of the exosomes [7,11]. Regarding protein expression, exosome marker proteins CD9, GAPDH and HSP90 were detected in exosome solutions prepared from the two colostral samples used in Experiment 1. Based on these results, we confirmed that exosomes were properly isolated by the chosen method.

### 3.2. Compositions of T Cells in Piglet PBMCs Cultured with Milk-Derived Exosomes

**Figure 2:**
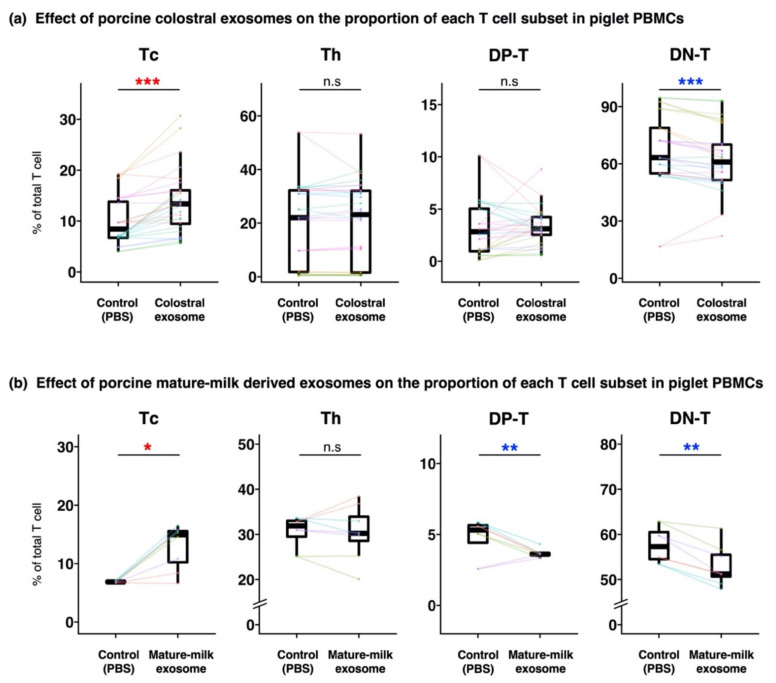
Effect of porcine milk-derived exosomes on the proportion of each T cell subset in piglet PBMCs. Piglet PBMCs were cultured with or without exosome solution isolated from colostrum and mature milk. The proportions of T cell subsets in PBMCs cultured with or without colostral exosome (**a**) or with or without mature-milk derived exosome (**b**), and analyzed by flow cytometry, were compared. The boxplot shows the individual datapoints (the means of quadruplicates of each culture) and their ranges, medians, and quartiles. Datapoints derived from the same piglet are connected by lines. Significance was tested using the Wilcoxon signed-rank test (* *p* < 0.05; ** *p* < 0.01; *** *p* < 0.001). The colors of the symbols indicate higher (red) and lower (blue) values in comparison with those of control. Additionally, (**a**) is based on the data obtained throughout Experiment 1–3. Boxplots for the data obtained from each of the experiments is shown in Figure S3.

The ratio of live cells was almost consistent irrespective of samples, and it was about 70%. The proportions of T cell subsets in PBMCs cultured with milk-derived exosomes were compared with those in the controls (T cell subsets in control PBMCs were added PBS instead of milk-derived exosomes). The proportions of Tc and DN-T cells were significantly higher and lower, respectively, in PBMCs cultured with colostral exosomes than in the control (Figure 2a, Figure S3). In Experiment 2, PBMCs were also cultured with exosomes derived from mature milk. They had significantly higher and lower proportions of Tc cells and DP-T cells and DN-T cells, respectively, than the control (Figure 2b).

**Figure 3:**
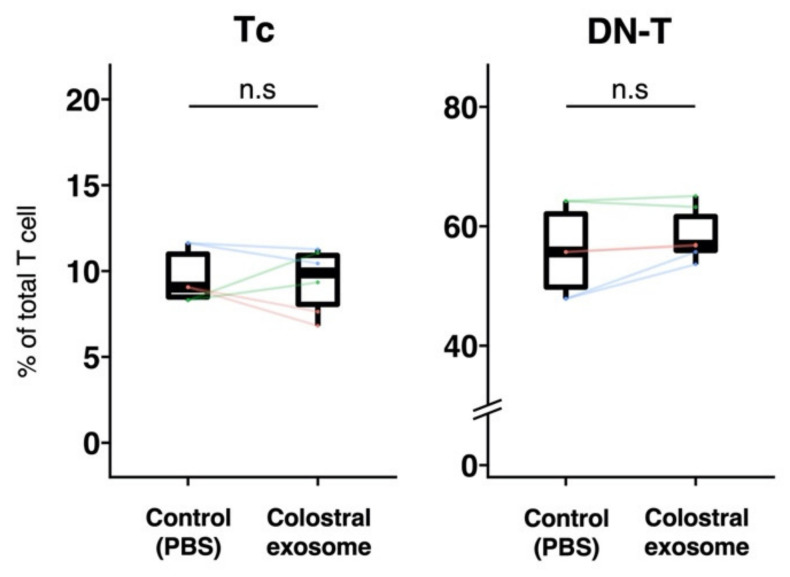
Effect of porcine colostral exosomes on the proportion of Tc cells and DN-T cells in Dynasore-treated PBMCs. To inhibit dynamin, PBMCs were treated with Dynasore hydrate and afterwards cultured with or without colostral exosomes. The proportions of Tc cells and DN-T cells were analyzed by flow cytometry three days post-incubation. The boxplot shows individual datapoints (the means of quadruplicates of each culture) and their ranges, medians, and quartiles. Datapoints derived from the same piglet are connected by lines. Significance was tested using the Wilcoxon signed-rank test.

In Experiment 2, the effect of milk-derived exosomes on the proportions of Tc cells and DN-T cells in Dynasore-treated PBMCs cultured with colostral exosome, was further evaluated. Compared with those of control, no significant differences in the proportions of Tc cells and DN-T cells in Dynasore-treated PBMCs cultured with exosomes were observed (Figure 3).

### 3.3. Concentration of IFN-γ, IL-2 and IL-12 in Culture Medium

**Figure 4:**
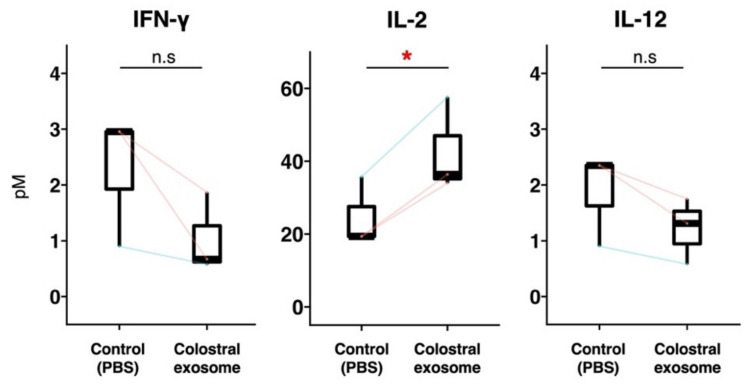
Concentrations of IFN–γ, IL–2 and IL–12 in culture media of piglet PBMCs. Piglet PBMCs were cultured with or without exosomes of colostrum from sows 5, 6 and 7. Three days post-incubation, the concentrations of IFN–γ, IL–2 and IL–12 in culture supernatants were measured using ELISA and these concentrations were then compared with those of PBMCs cultured with or without colostral exosome. The boxplot shows individual datapoints (the means of quadruplicates of each culture) and their ranges, medians, and quartiles. Datapoints derived from the same piglet are connected by lines. Significance was tested using the paired *t*-test (* *p* < 0.05).

In Experiment 3, to investigate the mechanism by which the proportion of Tc cells increased in the presence of exosomes, the concentrations of cytokines IFN-γ, IL-2 and IL-12 (which stimulate Tc cells) were measured. The concentration of IL-2 was significantly higher in the culture supernatant of PBMCs cultured with colostral exosomes than in that of the control (Figure 4). The concentrations of IFN-γ and IL-12 were non-significantly lower in exosomes-treated PBMCs than in the control. None of the evaluated cytokines were detected in the culture media collected immediately prior to the start of co-culturing (0 h).

## 4. Discussion

Mounting evidence reports that porcine colostral exosomes may contribute to the healthy growth and/or development of piglets [5,6,10]. In the present study, we evaluated the effect of porcine milk-derived exosomes, focusing on those from colostrum, on T cells in the peripheral blood of suckling piglets. Piglet PBMCs cultured with porcine colostral exosomes had higher and lower proportions of Tc cells and DN-T cells, respectively, than the control. It is well known that DN-T cells differentiate into Tc cells or Th cells [12]. In the present study, the population of each T cell subset was expressed as a relative percentage to the total T cell population. Thus, our results (i.e., higher Tc cell and lower DN-T cell proportions) may be interpreted in two ways: (1) colostral exosomes stimulated the differentiation of Tc cells from DN-T cells; and (2) colostral exosomes stimulated the proliferation/survival of Tc cells, resulting in a relative decrease in the proportion of DN-T cells. Previous studies reported that the proportion of Tc cells gradually increases in the peripheral blood of neonatal piglets until weaning [13,14]. Based on this fact, it can be reasonably assumed that, in the present work, porcine colostral exosomes were partly involved in the maturation of the immune system of suckling piglets.

To further explore the mechanism by which porcine colostral exosomes induced an increase in Tc cells, dynamin in PBMCs was inhibited with Dynasore and these cells were then cultured with colostral exosomes. No increase in Tc cells in Dynasore-treated PBMCs were observed. Three types of pathways through which an exosome mediates cell–cell communication have been reported: (1) membrane fusion, (2) endocytosis and (3) receptor binding [7,11]. Dynamin is an essential compound for cellular endocytosis. Thus, it is likely that inhibiting dynamin in PBMCs prevented endocytosis, paving the way to assume that exosomes likely helped increase the Tc cell population in PBMCs via endocytosis.

In Experiment 2, we investigated whether or not an increase in Tc cells was specifically triggered by porcine colostral exosomes. It was observed that, in PBMCs, not only porcine colostral exosomes but also those in mature milk increased and decreased the proportions of Tc and DN-T cells, respectively. Therefore, it was concluded that an increase in Tc cells was not a specific function of porcine colostral exosomes. It is widely accepted that the compositions of porcine colostrum and mature milk are different [15,16]. For example, Gu et al. [6] and Ferreira et al. [17] reported that, in sows, the microRNA and protein composition in colostral exosomes changes when observed at different time points, respectively. An analysis of the exosomal compositions of colostrum and mature milk was beyond the scope of the present study. However, our results seemed to suggest that the exosomal components that colostrum and mature milk shared were likely factors affecting the Tc cell population in PBMCs.

We explored the mechanism by which porcine colostral exosomes could induce an increase in the proportion of Tc cells in piglet PBMCs. A preliminary experiment in which DN-T cells were cultured with porcine colostral exosomes failed to show an increase in the proportion of Tc cells (data not shown). This result seemed to imply that milk-derived exosomes could help Tc cells survive and/or proliferate but not differentiate from DN-T cells. Therefore, in Experiment 3, as the indirect factors, we focused on cytokines affecting T cell activity. Supernatants resulting from culturing PBMCs with exosomes had a significantly higher concentration of IL-2 and a numerical but non-significant lower concentration of IFN-γ and IL-12, than the control. Although all three measured cytokines contribute to induce the survival/proliferation of Tc cells, they have different mechanisms of action [18,19,20]. Our results indicated that the milk-derived exosome affected the expression pattern of cytokines in PBMCs, leading to compositional changes in T cell subsets. Nonetheless, although it can be theorized that in the present study cytokine IL-2 production, at least indirectly, induced Tc cell survival/proliferation, the mechanism by which milk-derived exosomes induced an increase in Tc cells remained unclear. It must be mentioned that the number of samples used for cytokine measurements was limited in this study. Thus, possible effects of factors relating to this study such as age difference in piglets were not excluded, although we used the youngest 15-day-old piglet (Piglet 12) and the oldest 30-day-old piglet (Piglet 13). To better elucidate said mechanism, namely, the involvement of IL-2, using a larger sample size, and a comprehensive proteomic and transcriptomic analysis of the expression patterns of cytokines is recommended.

It is worth noting that, in the present study, there were no dam–litter relationships between sows and piglets. Hence, the effect of milk-derived exosomes on the Tc cell population observed in the present study was bloodline-independent.

## 5. Conclusions

The present study demonstrated that porcine colostral exosomes increased the proportion of Tc cells in the peripheral blood of suckling piglets. Stimulation via endocytosis of IL-2 production in PBMCs was theorized to be the possible underlying mechanism. Based on the present results, it can be inferred that porcine colostral exosomes were likely involved in the development of cellular immunity in suckling piglets.

## Figures and Tables

**Table 1:** List of piglet and sow combinations used for the culture of piglet PBMCs with milk-derived exosomes.

					Source of Milk-Derived Exosomes							
					Sow ID	Sow 1	Sow 2	Sow 3	Sow 4	Sow 5	Sow 6	Sow 7
Source of PBMCs					Breed ^1^	LW	LW	LW	LW	L	L	LW
Piglet ID	Breed ^1^	Location ^2^	Age	Sex	Location ^2^	Kyoto	Kyoto	Kanagawa	Kanagawa	Kanagawa	Kanagawa	Kyoto
Experiment 1												
Piglet 1	LWD	Kyoto	21 d	Female		a	a					
Piglet 2	LWD	Kyoto	20 d	Male		a	a					
Piglet 3	LWD	Kyoto	20 d	Female		a	a					
Piglet 4	LWD	Kagawa	29 d	Female		a	a					
Piglet 5	LWD	Kagawa	29 d	Female		a	a					
Experiment 2												
Piglet 6	LWD	Kanagawa	30 d	Female				a, c	a, c			
Piglet 7	LWD	Kanagawa	30 d	Female				a, b, c	a, b, c			
Piglet 8	LWD	Kanagawa	25 d	Male				a, b, c	a, b, c			
Piglet 9	LWD	Kanagawa	25 d	Female				a, b, c	a, b, c			
Experiment 3												
Piglet 10	LD	Kanagawa	30 d	Female						a	a	
Piglet 11	LD	Kanagawa	27 d	Female						a	a	
Piglet 12	LD	Kanagawa	15 d	Male						a, d	a, d	a
Piglet 13	LD	Kanagawa	30 d	Male						a	a	a, d

a: Piglet PBMCs were cultured with porcine colostral exosomes in total 28 combinations; b: Dynasore-treated PBMCs were cultured with porcine colostral exosomes in total 6 combinantions; c: Piglet PBMCs were cultured with porcine mature-milk exosomes in total 8 combinations; d: Measurement of the concentrations of IFN-γ, IL-2, and IL-12 in culture supernatants post-co-culture; ^1^ Breeds of piglets and sows: L, Landrace; W, Large White; D, Duroc; ^2^ Locations of the experimental farms: Kagawa, Kagawa University; Kanagawa, Azabu University; Kyoto, Kyoto Prefectural University.

## Data Availability

Data are available from the corresponding author.

## Supplementary Materials

The following are available online at https://www.mdpi.com/article/10.3390/ani12172172/s1. Figure S1: Representative image of flow cytometric analysis of T cell subsets; Figure S2: Whole blot images of Western blotting analysis; Figure S3: Effect of porcine milk-derived exosomes on the proportion of each T cell subset in piglet PBMCs in each experiment.
