# Supplementary material for: Effect of Porcine Colostral Exosomes on T Cells in the Peripheral Blood of Suckling Piglets
Source: Animals (Basel). 2022 Aug 24;12(17):2172. doi: 10.3390/ani12172172 (PMC9455021; doi:10.3390/ani12172172)
Supplement: Supplementary file 1 [file animals-12-02172-s001.zip › animals-1842242-supplementary.pdf]

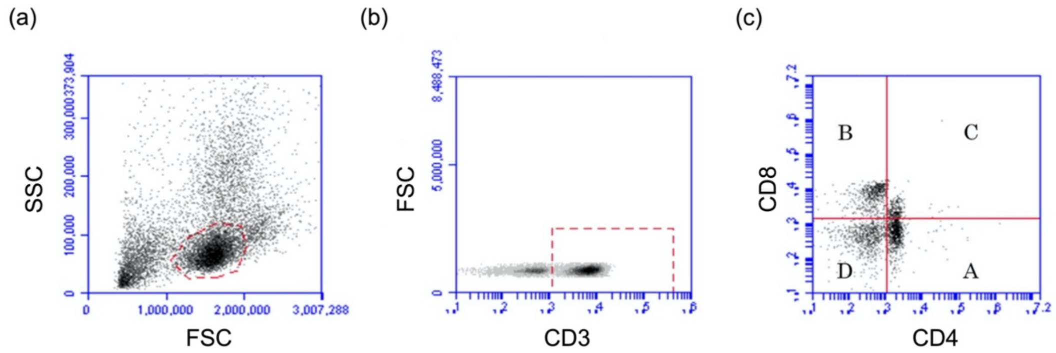

Figure S1. Representative images of the flow cytometric analysis of T cell subsets. First, based on size, lymphocytes were identified from peripheral mononuclear cells (red circle, a). Among lymphocytes, CD3 positive cells were then identified as T cells (red square, b). Based on the expression of CD4 and CD8, total T cells were sorted and then identified as follows: CD4+CD8- T cells, helper T (Th) cells (fraction A, c); CD4-CD8+ T cells, cytotoxic T (Tc) cells (fraction B, c); CD4+CD8+ T cells, double positive T (DP-T) cells (fraction C, c); CD4-CD8- T cells, double negative T (DN-T) cells (fraction D, c).

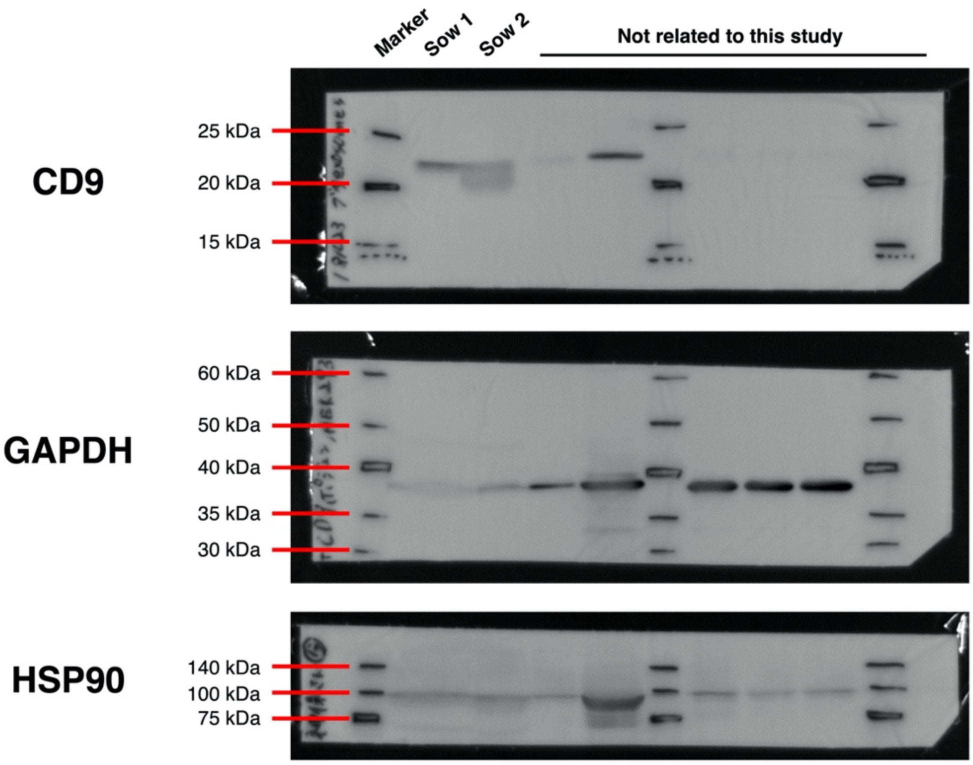

Figure S2. Whole blot images of western blotting analysis.

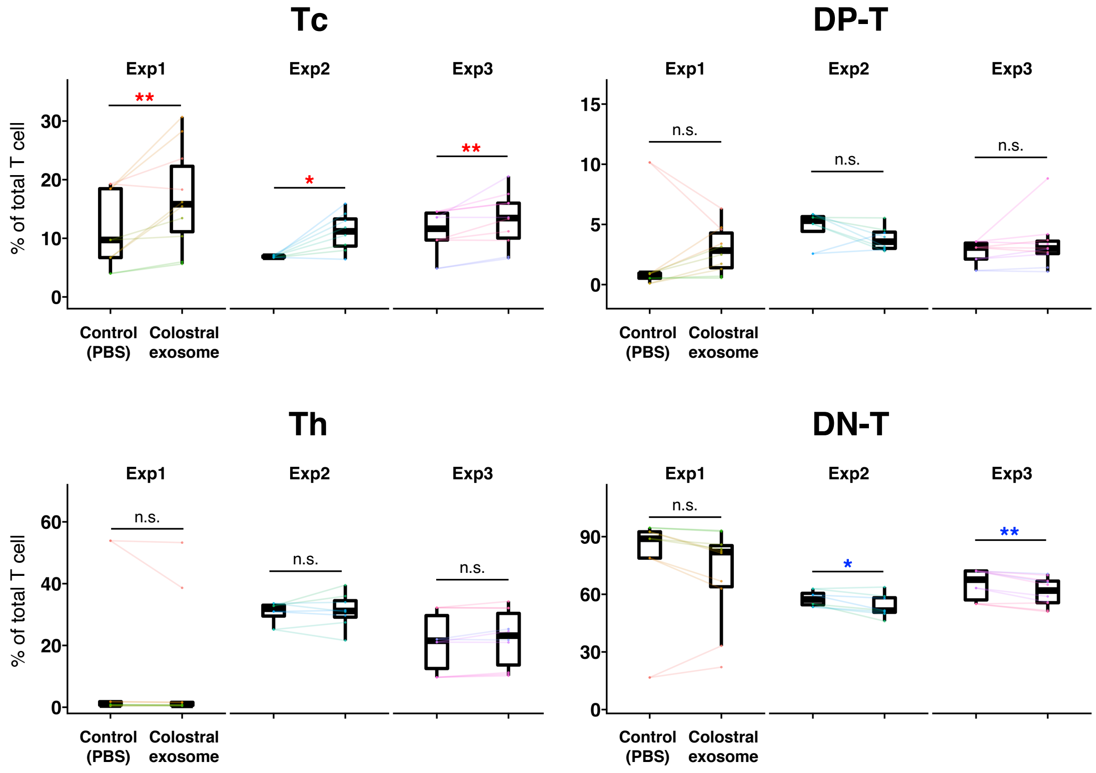

Figure S3. Effect of porcine milk-derived exosomes on the proportion of each T cell subset in piglet PBMCs in each experiment. The data presented in Figure 2a were separated by each experiment and visualized. Piglet PBMCs were cultured with or without exosome solution isolated from colostrum. See caption of Fig 2 for the details.
